# Altered methylations of *H19*, *Snrpn*, *Mest* and *Peg3* are reversible by developmental reprogramming in kidney tissue of ICSI-derived mice

**DOI:** 10.1038/s41598-017-11778-w

**Published:** 2017-09-20

**Authors:** Qitao Zhan, Xuchen Qi, Ning Wang, Fang Le, Luna Mao, Xinyun Yang, Mu Yuan, Hangying Lou, Xiangrong Xu, Xijing Chen, Fan Jin

**Affiliations:** 1 Department of Reproductive Endocrinology, Women’s Hospital, School of Medicine, Zhejiang University, Hangzhou, 310006 China; 2 Department of Neurosurgery, Sir Run Run Shaw Hospital, School of Medicine, Zhejiang University, Hangzhou, 310016 China; 3 Key Laboratory of Reproductive Genetics, Ministry of Education, Hangzhou, 310006 China

## Abstract

Although the prevalence of Intracytoplasmic sperm injection (ICSI) has increased year by year, there remains concern about the safety of these procedures because of reports of the increased risk for imprinting disorders. Previous research has demonstrated that gonadotropin stimulation contributes to an increased incidence of epimutations in ICSI-derived mice. However, the epimutations in ICSI offspring after removing the effect of gonadotropin stimulation and the possibility that epimutations are reversible by developmental reprogramming has not been investigated. Our study is the first to investigate the effect of ICSI itself on methylation and exclude the effect of superovulation using the kidney tissues from the adult and old mice. We found reduced methylation and up-regulated expression of the imprinted genes, *H19*, *Mest* and *Peg3*, in adult ICSI mice, but the above alterations observed in adult mice were not detected in old ICSI mice. At the *Snrpn* DMR, methylation status was not altered in adult ICSI-derived mice, but hypermethylation and correlated down-regulated expression of *Snrpn* were observed in old mice. In conclusion, ICSI manipulation and early embryo culture resulted in alterations of methylation in differentially methylated region of *H19*, *Mest*, *Peg3* and *Snrpn*, and the alterations were reprogrammed by developmental reprogramming.

## Introduction

Intracytoplasmic sperm injection (ICSI), one type of assisted reproductive technologies (ART), was firstly presented as a successful method over two decades and nowadays has become a widely-accepted medical treatment for male infertility. With the rising utilization of ICSI, several concerns of the progeny, therefore, have been raised. In theory, the ICSI technology is in the absence of natural selection of sperm; any structural damage to the oocyte produced by the injection or even the transfer of heterologous substances, such as culture solution, that would not been passed on to the progeny normally, might increase the potential for epigenetic errors, or epimutations. Meanwhile, gonadotropin stimulation, and *in vitro* culture of germ cell and resulting early embryo followed by transfer of embryo in the ICSI program, might also induce a higher risk of certain developmental abnormalities or disease phenotypes in offspring^1^.

Indeed, several studies indicated that children conceived by ICSI are at an increased risk for birth defects^2–5^ and imprinted diseases^6–9^. Hansen *et al*. compared 1138 infants born following the use of ART (301 infants with ICSI and 837 with *in vitro* fertilization) with 4000 naturally conceived infants, suggesting infants conceived with the use of ART had twice as high a risk of a major birth defect as naturally conceived infants^10^. Meanwhile, a nationwide German cohort study showed the relative risk of major malformation at birth was 1.44 in pregnancies established after ICSI, as compared with spontaneously conceived pregnancies^11^. Besides, systemic reviews suggested that children born following ICSI are at a higher risk of autism than the general population^12^. Nevertheless, the mechanisms behind the excess of malformation are still unclear. The parental genetic factors and the ICSI program itself are the possible explanations.

Germ cells and early embryos are unique, because they must undergo extensive epigenetic reprogramming to reestablish developmental potency during each generation^13^. Firstly, on every reproductive cycle, genomic imprints in the parental gametes are erased. Then, imprints in the immature germ cells of the developing embryo were reestablished according to their fate either as male or female gametes. Finally, imprints were maintained by both the preimplantation (when the rest of the genome is demethylated) and postimplantation development. Therefore, genomic imprints are dynamically changing during the developments of germ cell and embryo^14^. ICSI treatment, which is operated in the timing of *in vitro* culture of germ cell and fertilization and early embryo development, may interfere with the acquisition and maintaining of genomic imprints. The modification of imprinting center region (ICR) or differentially methylated region (DMR) during ICSI treatment were proofed to might contribute to birth defects or imprinted diseases^15^.

A possible excess of genitor-urinary defects, cardiovascular defects, gastrointestinal defects and nervous defects was shown in children conceived by ICSI^16–18^. Until now, the long-term effects of ICSI treatment have not been fully evaluated, especially in the adult and old ones, both because most individuals born through ICSI are still under 25-year-old and because research on potential effects in human or animal offspring is still ongoing. The more important thing is that, among the studies devoted to finding the mechanisms behind the association between diseases and ICSI treatment, most of them focus on the cardiovascular, gastrointestinal and nervous system defects by using placenta, liver, muscle and brain tissues^1,19–21^, few have focus on the urinary system by using kidney tissue.

**Figure 1:**
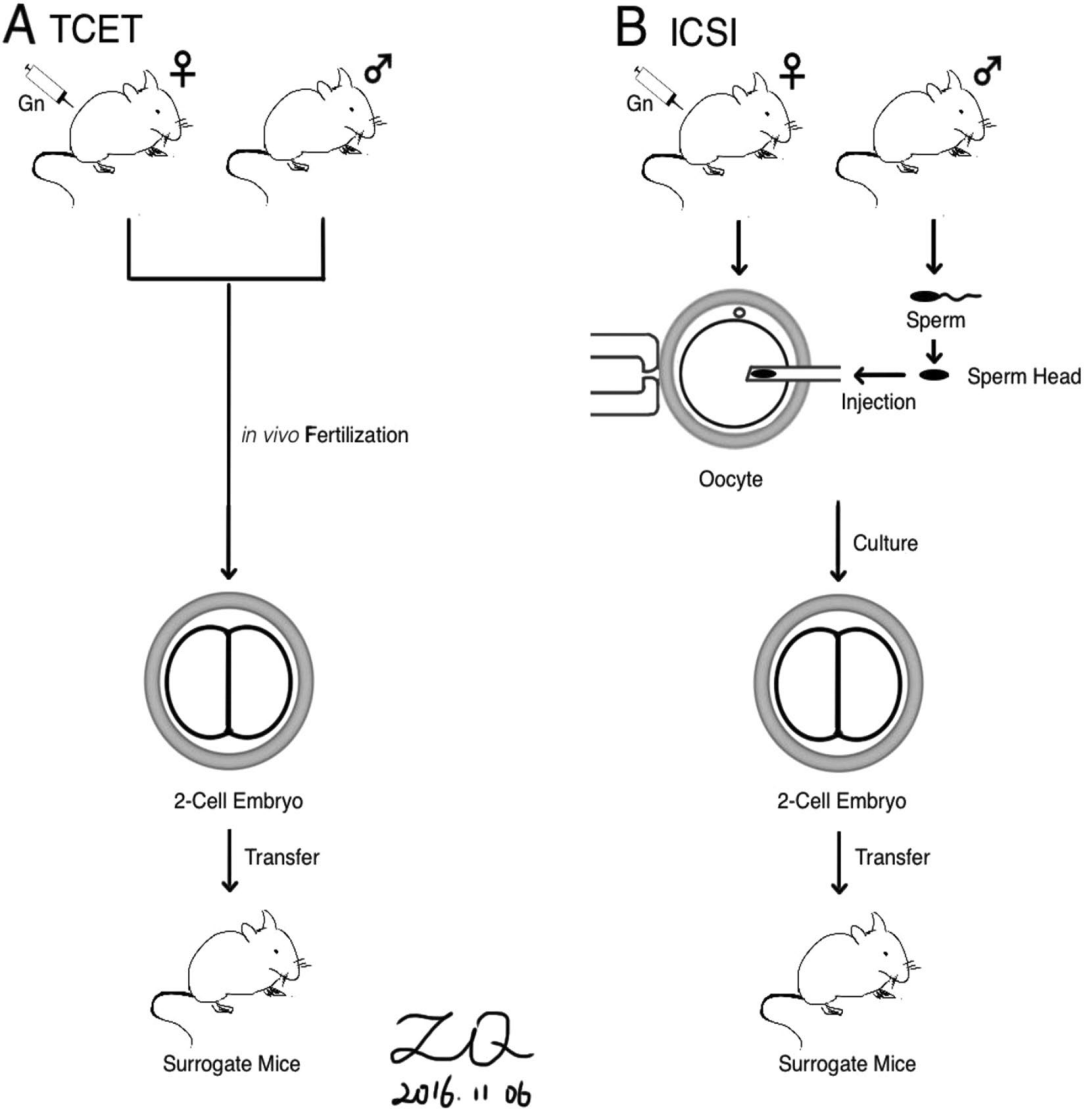
Schematic comparison of the TCET and ICSI procedures. (**a**) The TCET group is established by *in vivo* fertilization after gonadotropin stimulation. After 2-cell embryo is obtained from the oviduct, the embryo is subsequently transferred into a pseudo-pregnant mouse. (**b**) The ICSI mice also begin with the injection of gonadotropin and end with transferring 2-cell embryo into surrogate mice. A primary difference between the two procedures is the manipulation and injection of occytes and culture for ICSI, while only *in vivo* fertilization for TCET. (Drawn by authors Q. Z and X. Q).

Therefore, in our study, we examined kidney tissues, which were seldom been reported, from both the adult and the old mice. ICSI program includes four procedures, gonadotropin stimulation, manipulation and injection of occytes and culture followed by transfer of embryos. Then, which aspect or aspects directly contributes to the genesis of defects in the offspring? Previous observations suggested gonadotropin stimulation contributes to an increased incidence of epimutations in ICSI-derived mice^1^ and alteration of fatty acid metabolism^20,22^. To exclude the effect of gonadotropin stimulation, we analyzed ICSI-derived mice with mice derived by two-cell embryos transfer (TCET) instead of traditionally control mice which were derived by natural mating^23^. A key difference between ICSI and TCET mice is that manipulation and injection of oocytes and culture of early embryo during the ICSI program are removed in the TCET process and replaced by *in vivo* fertilization (Fig. 1). We focused on paternally imprinted gene *H19*, and maternally imprinted genes *Snrpn*, *Mest*, and *Peg3*, which were reported kidney disease related genes^24–28^, and measured the DNA methylation and expression of these genes at their DMRs. In this research, we tried to elucidate the underlying epigenetic mechanisms for these defects and provided evidences for the safety of ICSI.

## Results

### Alterations of methylation were detected in kidney tissues of adult ICSI mice

**Figure 2:**
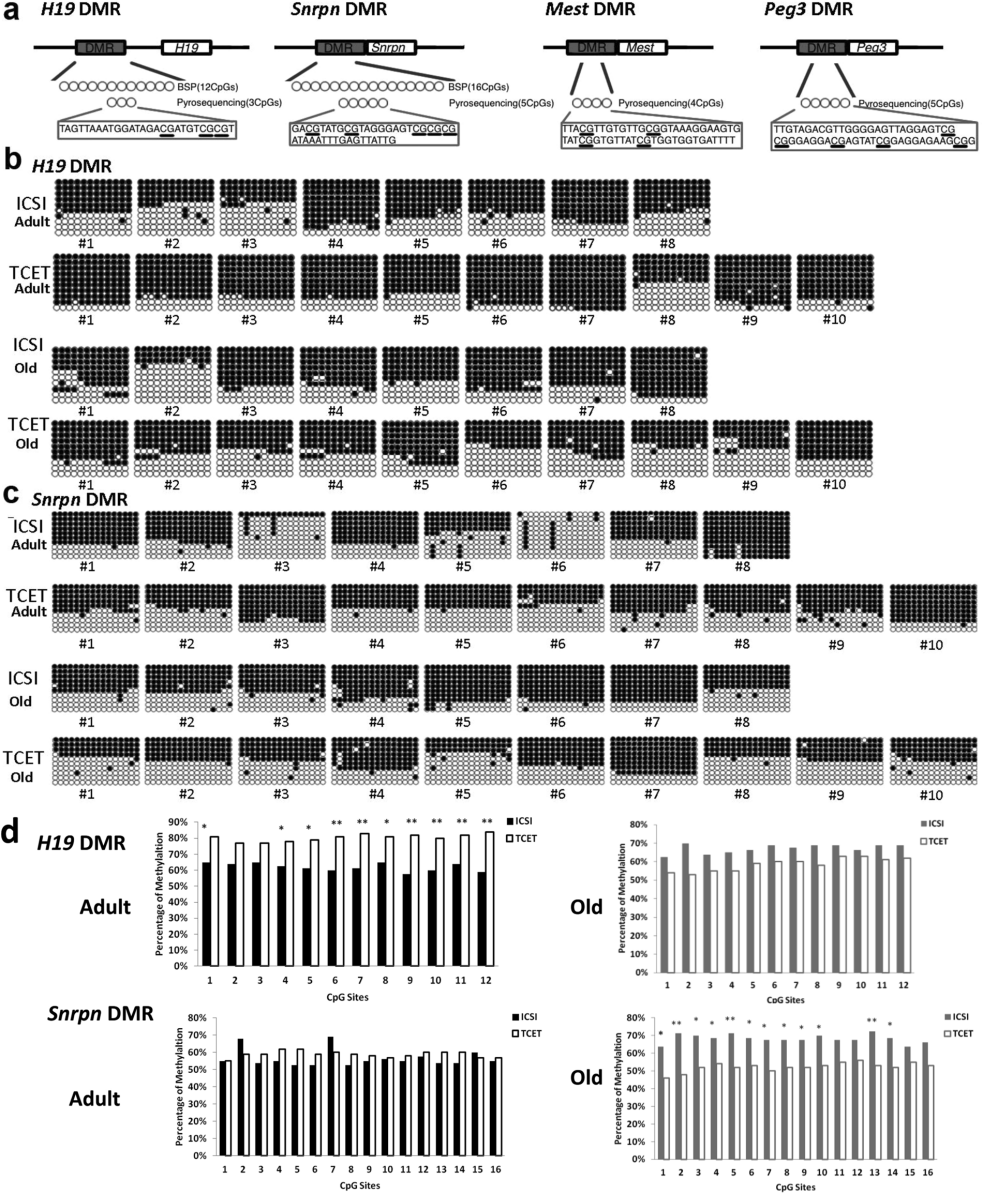
DNA methylation of the *H19* and *Snrpn* DMRs in kidney tissues of adult and old ICSI-derived and TCET conceived mice. (**a**) Schematic representation of the differential methylated regions analyzed by bisulfite sequencing PCR (BSP) or pyrosequencing or both of them. The target sequences examined by pyrosequencing were in the boxes and CpG sites were underlined. (**b**) Methylation profiles by BSP (12 CpG sites) for the *H19* DMR from ICSI-derived and TCET conceived mice. (**c**) Methylation profiles by BSP (16 CpG sites) for the *Snrpn* DMR from ICSI-derived and TCET conceived mice. (**d**) Statistical methylation analysis of *H19* DMR and *Snrpn* DMR (Pearson chi-square test was used for the analysis: **p < 0.01; *p < 0.05).

**Figure 3:**
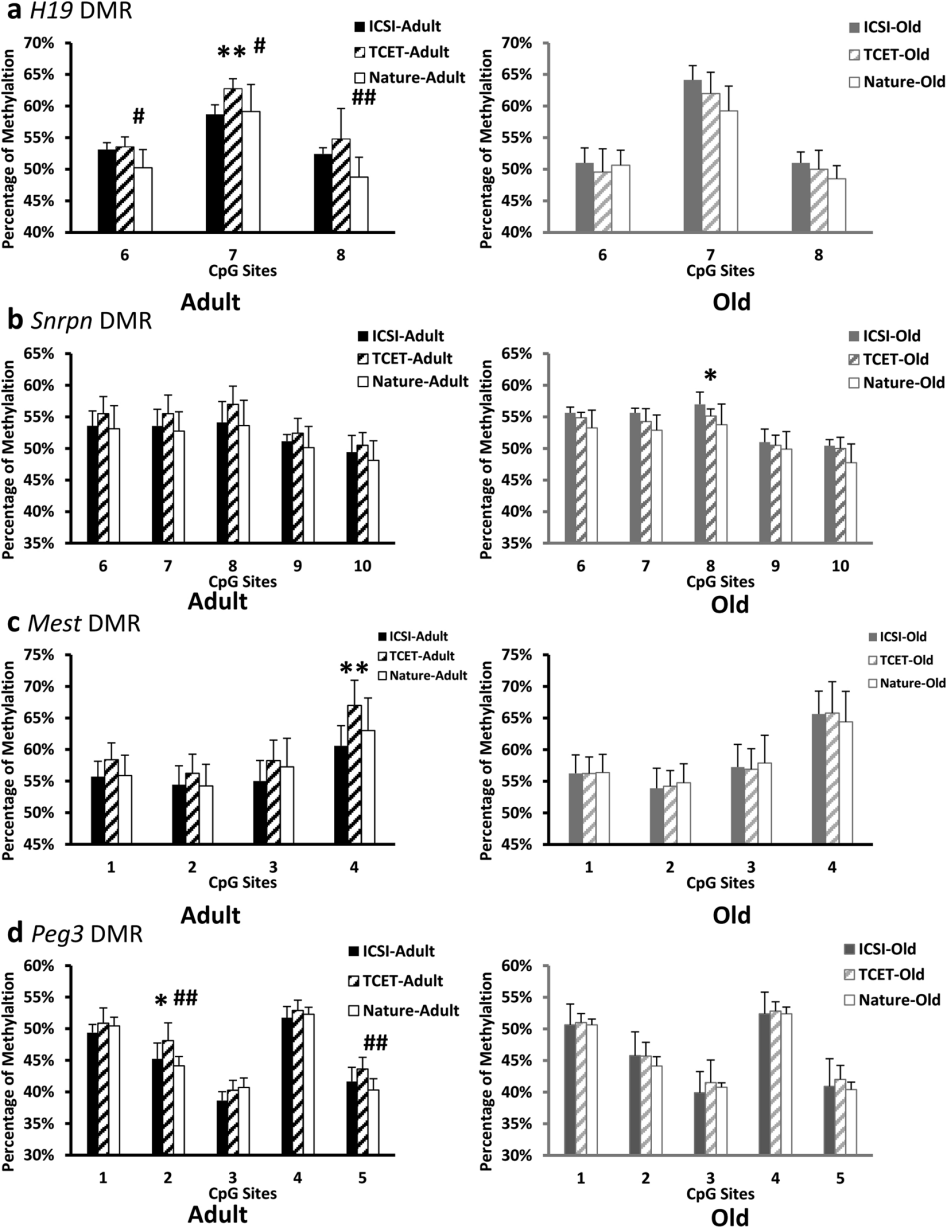
Pyrosequencing analyses of the methylation profiles from ICSI-derived, TCET conceived and natural mating mice of adult and old age. There are 3 CpG sites for the *H19* DMR (**a**), 5 CpG sites for the *Snrpn* DMR (**b**), 4 CpG sites for the *Mest* DMR (**c**) and 5 CpG sites for the *Peg3* DMR (**d**) (t-test was used for the analysis. ICSI versus TCET: **p < 0.01; *p < 0.05. TCET versus Nature: ^##^p < 0.01; ^#^p < 0.05). The pyrogram of pyrosequencing of one sample in each gene and each group was shown in Supplementary Figure S1.

Kidney tissues from 16 ICSI-derived mice (8 adults and 8 olds) and 20 TCET-derived mice (10 adults and 10 olds) were used to analyze the DNA methylation and expression of the imprinted genes. DNA methylation level of imprinted gene *H19* was first examined by Bisulfite sequencing PCR (BSP) in DMR island 3 containing 12 CpG sites. Compared with mice conceived by TCET, mice conceived through ICSI exhibited hypomethylation at all 12 CpG sites, ten of which (the 1st site and site from the 4th to the 12th) showed significantly hypomethylated level (p < 0.05) (Fig. 2d). Next, to verify the result of BSP, we used bisulfite pyrosequencing assay to examine 3 CpG sites of the region previously analyzed by BSP (Fig. 2a). Similar differential methylation was presented. Three CpG sites that showed relative hypomethylation in ICSI mice based on BSP also displayed reduced methylation when kidney tissues were examined by pyrosequencing (Fig. 3a). In addition, the 7th CpG site was significantly hypomethylated in ICSI mice (58.71% ± 1.49% in ICSI mice vs. 62.77% ± 1.56% in TCET mice, p < 0.01).

**Figure 4:**
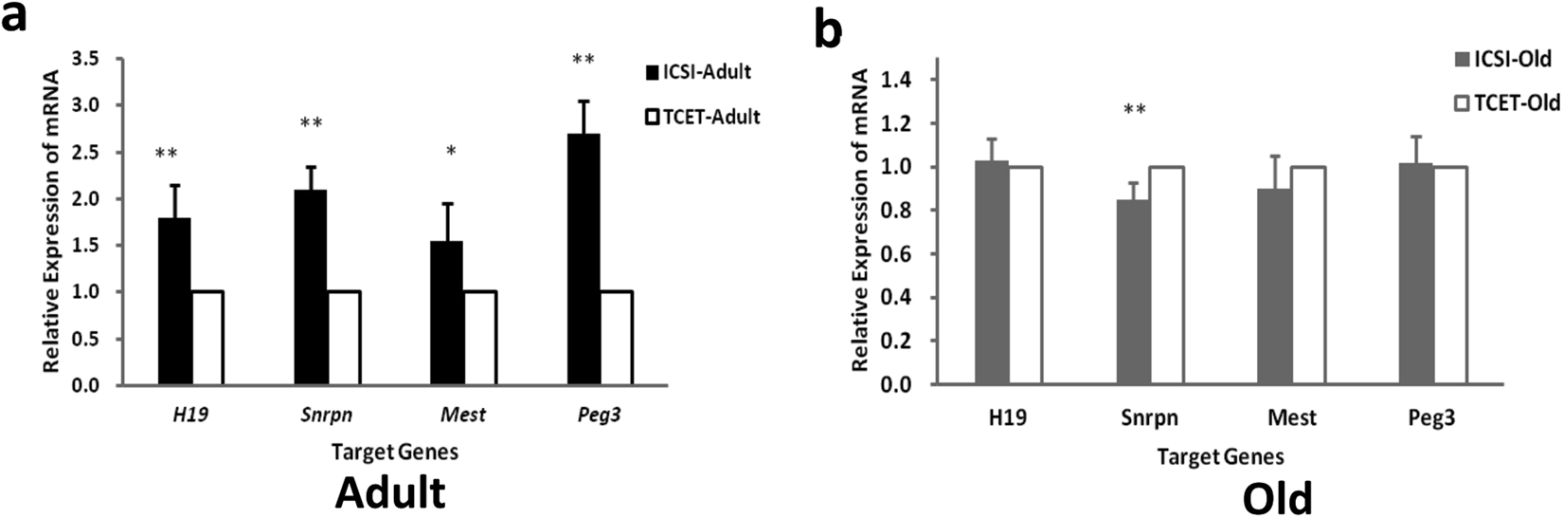
Relative expression of mRNA of the imprinted genes in kidney tissues of ICSI-derived and TCET-derived mice. (**a**) and (**b**) are the results of adult and old mice (**p < 0.01; *p < 0.05).

We used real-time RT PCR to quantify expression of *H19* in kidney tissues from the same ICSI conceived and TCET-derived mice (Fig. 4a). Significantly up-regulated expressions were showed in ICSI mice, suggesting that the hypomethylation actually represented a valid change.

Maternally imprinted gene *Snrpn* in the same adult mice conceived either by ICSI or by TCET was also analyzed. We performed BSP to examine a position that contained 16 CpG sites in the *Snrpn* DMR. Over 80% (13 out of 16) CpG sites displayed reduced methylation in ICSI-derived mice when compared with TCET mice (Fig. 2d). The aberrant hypomethylation in ICSI mice also represented a valid alteration. There were two reasons: firstly, the mice derived by ICSI showed up-regulated expression compared with mice derived through TCET (Fig. 4a); secondly, the hypomethylation was also detected by bisulfite pyrosequencing (Fig. 3b).

DNA methylation at the second maternally imprinted gene *Mest* was also analyzed in the same ICSI mice and TCET mice. The DMR region containing 4 CpG sites was examined only by bisulfite pyrosequencing because of the consistent results of the previous genes obtained through BSP and pyrosequencing (Figs 2a,d and 3a and b). TCET mice exhibited differential methylation and the average methylated percentage was 60%. However, ICSI-derived mice displayed reduced methylation (on the 4th CpG sites: 60.57% ± 3.21% in ICIS mice vs. 67.00% ± 3.96% in TCET mice, p < 0.01) (Fig. 3c) and a correlated up-regulated expression of mRNA from the ICSI mice (Fig. 4a).

Finally, we analyzed the imprinted *Peg3* gene, which is paternally expressed in kidney tissue. The region examined by pyrosequencing contained 5 CpG sites (Fig. 2a). TCET mice displayed differential methylation and the average methylation was 46%. While mice born through ICSI showed relative hypomethylation (on the 2nd CpG sites: 45.25% ± 2.49% in ICSI mice vs. 48.13% ± 2.80% in TCET mice, p < 0.05) (Fig. 3d) and also displayed a correlated up-regulated expression of mRNA compared with mice born through TCET (Fig. 4a).

### Alterations of methylation might be reversible in kidney tissues of old ICSI mice

To determine whether methylation is dynamic during the development, we analyzed methylation and expression of the same imprinted genes in old mice born through ICSI and TCET. Surprisingly, we found hypermethylation at the maternally imprinted *Snrpn* DMR in the old ICSI-derived mice (p < 0.01) instead of hypomethylated methylation in the adult ICSI-derived mice when compared with the corresponding TCET mice (Fig. 2d). We also used bisulfite pyrosequencing assay to examine 5 CpG sites of the region previously analyzed by BSP. Similar differential methylation was presented. CpG sites that showed relative hypermethylation in ICSI old mice based on BSP also displayed a raised methylation when kidney tissues were examined by pyrosequencing (Fig. 3b). In addition, the 8th CpG site was significantly hypermethylated in ICSI mice (57.70%% ± 1.50% in ICSI mice vs. 54.10% ± 1.05% in TCET mice, p < 0.01).

The hypermethylation also represented a valid change, because a significantly down-regulated expression of *Snrpn* was shown in old ICSI mice (Fig. 4b). Nevertheless, methylation at the paternal imprinted *H19* DMR, maternal imprinted *Mest* DMR and *Peg3* DMR were examined, alterations of methylation that displayed in adult mice were not detected in old mice produced by ICSI compared with mice produced through TCET (Figs 2d and 3a,c,d). Similar expression of the above three imprinted genes *H19*, *Mest*, and *Peg3*, respectively, were also displayed in ICSI mice and TCET mice (Fig. 4b). Thus, it appears that DNA methylation can be reversible and always with correlated expression.

### Hypermethylations were detected in kidney tissues of adult TCET mice and these alterations were reduced in kidney tissues of old TCET mice

De Waal E. *et al*. reported that gonadotropin stimulation contributed to an increased incidence of epimutations in ICSI-derived mice^1^. In our study, we evaluated the effects of gonadotropin stimulation and embryo transfer procedures by comparing the TCET mice and the natural mating (NM) mice. Hypermethylation was detected at the paternal imprinted *H19* DMR, maternal imprinted *Snrpn* DMR, *Mest* DMR and *Peg3* DMR (Fig. 3a–d). The 6th to the 8th CpG sites at *H19* DMR were significantly hypermethylated in TCET mice compared with the NM control (the 6th CpG site: 53.56%% ± 1.51% in TCET mice vs. 50.25% ± 2.87% in NM mice, p < 0.05; the 7th CpG site: 62.78%% ± 1.56% in TCET mice vs. 59.13% ± 4.29% in NM mice, p < 0.05; the 8th CpG site: 54.78%% ± 4.84% in TCET mice vs. 48.75% ± 3.15% in NM mice, p < 0.01). Besides, raised methylations of the 2nd and the 5th CpG sites at *Peg3* DMR were also shown significant (the 2th CpG site: 48.13%% ± 2.80% in TCET mice vs. 44.14% ± 1.46% in NM mice, p < 0.01; the 5th CpG site: 43.63%% ± 1.85% in TCET mice vs. 40.29% ± 1.80% in NM mice, p < 0.01).

Similarly, we analyzed methylation of the four imprinted genes in old mice born through TCET or conceived naturally. The significantly raised methylations that found in the adult TCET mice were not existed in the old TCET mice compared with the NM control (Fig. 3a–d). Therefore, alterations of DNA methylation produced by gonadotropin stimulation and embryo transfer procedures might be reduced during the development.

## Discussion

Although the prevalence of ICSI has increased to about two-thirds or even higher of ART in some regions of the world^29^, there remains concern about the safety of this procedure because of reports of the increased risk for imprinting disorders. Of particular concern is the possibility that manipulations used in ICSI procedures might disrupt the normal acquisition and maintaining of genomic imprints during the development of germ cell and embryo. A systematic review of imprinting disorders in children conceived by ART demonstrated an increase in imprinted disorders, but evidence for an association between ART and methylation was still insufficient^30^. Studies in animals demonstrated that gonadotropin stimulation contributed to an increased incidence of epimutations in ICSI-derived mice^1^. However, few studies have reported the epimutations in ICSI offspring after removing the effect of gonadotropin stimulation. Besides, the possibility that epimutations are reversible by developmental reprogramming has not been investigated.

Our study is the first to investigate the effect of ICSI itself on methylation and exclude the effect of superovulation by using the kidney tissues from the adult and old mice. To exclude the effect of gonadotropin stimulation, we analyzed mice derived by TCET as control group (Fig. 1). Manipulation and injection of occytes and culture during the ICSI program were removed during the TCET process and replaced by *in vivo* fertilization. We found reduced methylation and up-regulated expression of imprinted genes in adult ICSI mice when compared with age-matched TCET mice, whereas in old ICSI-derived mice, hypermethylation at the *Snrpn* DMR and correlated down-regulated expression of *Snrpn* were observed when compared with the corresponding TCET mice. In addition, alterations of *H19*, *Mest*, and *Peg3* methylation that observed in adult mice were not detected in old ICSI mice.

The effect of superovulation and embryo transfer procedures was also evaluated in kidney tissues. Hypermethyaltions were detected after the above procedures in the TCET offspring, which was consistent with the result of previous study^1^. And these alterations were reduced in the old mice, revealing that the hypermethylations might be corrected during the development. However, the TCET mice were surrogated by ICR mice instead of C57BL/6 J mice, so we could not eliminate the effect of maternal environment on the offspring.

Human studies reported higher methylation percentage of *H19*, *Snrpn*, *Mest* and *Peg 3* in ART children than in naturally conceived children^31–33^. A meta-analysis of data from relevant reports revealed that the weighted mean difference (95 percentage confidence intervals) in percentage of methylation between IVF/ICSI versus spontaneously conceived were as follows: *H19*: −0.46(−1.41, 0.49), *SNRPN*: −0.55(−1.55, 0.46), *MEST*: −0.47(−2.07, 3.01) and *PEG3*: −0.24 (−1.72, 1.24), suggesting hyper-methylated DNA in ART IVF/ICSI children^30^. Our study excluded the effect of ovarian stimulation to the offspring, and founded hypo-methylated DNA in the ICSI adult when compared with TCET offspring, revealing the hypermethylation in ICSI children might be resulted from ovarian stimulation rather than ICSI manipulation itself.

Animal studies showed different effect of ART on different tissues, especially between the embryonic and extra-embryonic tissue. For instance, Fauque *et al*. showed that IVF and embryo culture resulted in hypomethylation of *H19* DMR in placenta tissue, but not in fetal tissue^34^. Similarly, Mann *et al*. found loss of methylation at *H19* DMR and *Snrpn* promoter in the placenta but not, or to a lesser extent, in fetus^35^. However, because of ethical question, human studies examined tissues easily obtained or relatively noninvasive. Tissues examined included peripheral blood, placenta, umbilical cord blood, buccal swabs and amnion/chorion. Thus, research about the effect of ART on different tissues was in desperate need.

Urogenital abnormality was one of the most common abnormalities of ART children, and *H19*, *Mest*, *Peg3*, and *Snrpn* were the related imprinting genes. In previous research, we had found that the methylation and expression of *H19* in IVF offspring was changeable during the postnatal periods^36^. In this study, we found ICSI manipulation and early embryo culture really lead to methylation change of the above genes. More importantly, the methylation change was either reprogrammed or repaired during the development, and always with correlated mRNA expressions.

## Methods

### Animals

C57BL/6J female (6–8 weeks old) and male mice (8–10 weeks old) were used in this study to generate 16 mice conceived through the use of ICSI and 20 mice created through TCET. Kidney tissues were obtained from either adults (10 weeks old) or olds (1.5 years old), and then were used to analyze DNA methylation and expression of imprinted genes. For embryo transfer, both surrogated mothers (6–8 weeks old) and vasectomized males (8–10 weeks old) were ICR outbred mice.

### Statements of approval and accordance

All methods were reviewed and approved by Zhejiang University Animal Care Committee, and all experiments were performed in accordance with the Institutional Guidelines for Animal Experiments (No. ZJU2009101007Y).

### ICSI

ICSI was performed according to Wang and de Waal^1,21^. C57BL/6J female mice was subjected to hormone stimulation by consecutive injections of 7.5IU pregnant mareserum gonadotropin (PMSG, Organon) and 7.5 IU human CG (hCG, Organon) administered 46–48 hours apart. Approximately 13–15 hours after the injection of hCG, cumulus-oocyte complexes (COC) were collected from the oviducts. To remove cumulus cells, COC were treated with modified human tubal fluid medium (mHTF, Irvine). Cauda epididymal sperms of C57BL/6J male were suspended in warmed mHTF for 10 minutes, and a drop of sperm suspension was then mixed with polyvinylpyrrolidone (PVP, Irvine) in mHTF. The head of a single sperm was detached from the tail using a Piezo-driven pipette (PrimTech, Japan), and then injected into each oocyte by a micromanipulator (Narishige, Japan). Resulting zygotes were cultured for 24 hours to obtain two-cell stage embryos that were transferred into surrogate mice.

### TCET

C57BL/6J female mice were caged with males of the same strain after stimulated by consecutive injections of PMSG and hCG as described in ICSI group. Female mice with a vaginal plug were separated from the males on day 2. Forty-four hours later, two-cell stage embryos were obtained from the oviducts and then were transferred into pseudo-pregnant surrogate mothers.

### Embryo Transfer

All embryo transfers were performed with embryos at two-cell stage. Embryos derived from ICSI and TCET were transferred into the oviducts of ICR pseudo-pregnant mice (day 0.5) that had been mated with vasectomized ICR males the previous night. Newborn pups were obtained three weeks on average after embryo transfer. Sixteen mice conceived through the use of ICSI (8 adults and 8 olds) and 20 mice created through TCET (10 adults and 10 olds) were sacrificed to obtain kidney samples, which were then used to analyze the DNA methylation and expression of imprinted genes. Table 1 showed that body weights and weights of kidney tissues were similar between mice produced by TCET and mice conceived through ICSI (p > 0.05).

**Table 1:** Body weights and weights of kidney tissues in ICSI mice and TCET mice (Mean ± SD).

Weights (g)	Adult		Old	
	ICSI	TCET	ICSI	TCET
Body	25.00 ± 3.03	26.16 ± 3.78	24.73 ± 2.60	25.04 ± 3.56
Kidney	0.40 ± 0.07	0.38 ± 0.05	0.44 ± 0.05	0.40 ± 0.07

### DNA Methylation Analysis

DNA was isolated from kidney tissue by using proteinase K digestion followed by phenol-chloroform extraction and ethanol precipitation (SangonBiotech) at all the time points.

### Bisulfite sequencing PCR

Bisulfite sequencing PCR (BSP), which has served as the “gold standard” for measuring DNA methylation, was performed on genomic DNA (2 µg) by using the EpiTect Bisulfite kit (Qiagen) following the manufacturer’s instruction. Bisulfite-converted DNA (200 ng) was amplified by using PCR for *H19* and *Snrpn* DMRs. MethPrimer software^37^ was used to identify CpG islands and to design primers. For the *H19* DMR, the primer sequences were as follows: forward primer 5′-TTTTTGGGTAGTTTTTTTAGTTTTG-3′, reverse primer 5′-GTTTAATAAAGGGATTAGGTATTTGTGT-3′ (Wang et.al, 2012); and 12 CpG sites were analyzed (Fig. 2a). For the *Snrpn* DMR, the primer sequences were as follows: forward primer 5′-AATTTGTGTGATGTTTGTAATTATTTGG-3′, reverse primer 5′-TAAAATACACTTTCACTACTAAAATCC-3′; and 16 CpG sites were analyzed (Fig. 2a). The reaction conditions were as follows: predenaturation at 94 °C for 5 minutes, followed by 20 amplification cycles at 94 °C for 30 seconds, 60 °C to 50 °C (decreasing by 0.5 °C in each cycle) for 45 seconds, and 72 °C for 45 seconds, then a final extension and annealing step at 72 °C for 10 minutes^21^. Amplified products were gel purified by using NucleoSpin Extract II kit (Macherey-Nagel) according to the manufacture’s instruction. The purified products were ligated into the pMD 19-T Simple Vector (TaKaRa), and then transformed into *Escherichia coli* DH-5α competent cells and plated on LB plates with ampicillin (100 mg/mL) and incubated overnight at 37 °C. A single strain was inoculated into 2 mL LB liquid medium containing ampicillin (100 mg/mL) and grown overnight at 37 °C. Products were amplified using primer BcaBEST (TAKARA) which was specific for pMD 19-T Vector; and at least 10 clones from each sample for each imprinted genes with the expected band size were sequenced. The number of methylated CpGs (mCpGs) and unmethylated CpGs were recorded as previously described^21^. The percentage of overall mCpGs in each individual sites was calculated.

### Bisulfite Pyrosequencing

To reduce the variability in the methylation of CpG sites by BSP that possibly due to the additional bacterial cloning step, we further assessed DNA methylation profiles of the *H19* and *Snrpn* DMRs by using next-generation sequencing technology and performed custom pyrosequencing assays on 3 and 5 CpG sites, respectively, of the region previously analyzed by BSP (Fig. 2a). The results obtained through both methods were consistent (**Results part**). Because of the significant labor involved in BSP and the confirmed results of pyrosequencing, our study was limited to examining methylation of *Mest* and *Peg3* DMRs only by pyrosequencing. The sequencing primer for bisulfate pyrosequencing was designed using Pyrosequencing Assay Design Software (Qiagen). Primer sequences are shown in Table 2. The conditions of amplification were the same as BSP. The biotinylated primer was used to facilitate extraction of single-stranded DNA templates by using streptavidin-coated Sepharose Beads (Amersham) according to manufacturer’s instruction. Biotinylated PCR product (10 µl) was used for pyrosequencing with the sequencing primer. For the *H19* DMR, the sequencing primer was 5′-TTGTGTAGATTTGGTTATAG-3′ and 3CpG sites were analyzed. For the *Snrpn* DMR, the sequencing primer was 5′-TATGTGTAGTTATTGTTTGG-3′ and 5 CpG sites were analyzed. For the *Mest* DMR, the sequencing primer was 5′-GTAGTTTTTGGTATGTGGA-3′ and 4 CpG sites were analyzed. For the *Peg3* DMR, the sequencing primer was 5′-ACCCAAAATAAACATCTCT-3′ and 5 CpG sites were analyzed (Fig. 2a). Bisulfite pyrosequencing was performed using the PyroMark Q24 ID System (Qiagen) and the PyroMark Gold Q24 reagents kit (Qiagen). The degree of methylation at each CpG site was quantified with Pyro Q-CpG software (Biotage) and determined by dividing the incorporation of cytosine by incorporation of cytosine plus thymine (%C = C/C + T) at each site. The percent methylation was calculated by averaging the degree of methylation at each of the CpG sites analyzed.

**Table 2:** Primer sequences used for bisulfite pyrosequencing.

Gene (Position)	Primer Sequences	Product Size (bp)
*H19* DMR island3	(Forward) 5′-TTTTTGGGTAGTTTTTTTAGTTTTG-3′	211
(chr7:142580208-142580418)	(Reverse) 5′-biotin-ACACAAATACCTAATCCCTTTATTAAAC-3′	
*Snrpn* DMR	(Forward) 5′-TTTTGGTAGTTGTTTTTTGGTAGG-3′	240
(chr7: 60005020-60005259)	(Reverse) 5′-biotin-CACAAACCCAACTAACCTTCC-3′	
*Mest* DMR	(Forward) 5′-TTTAGGGTGTTTGTATTGTGATTG-3′	181
(chr6: 30737310-30737490)	(Reverse) 5′-biotin-TCACATAAAATAAACCAAAATCACC-3′	
*Peg3* DMR	(Forward) 5′-ACCAACCCAAAATAAACATCTCT-3′	115
(chr7: 6730345-6730459)	(Reverse) 5′-biotin-AGAGGATTTTGATAAGGAGGTGT-3′	

### mRNA Expression of Imprinted Genes

#### RNA Extraction and Reverse Transcription (RT)

Total RNA was extracted from kidney tissues using a Trizol-based protocol. In brief, 10 mg kidney tissue was homogenized in 1 ml Trizol (Invitrogen). After adding chloroform, RNA was isolated from the aqueous phase according to the manufacturer’s instructions (Invitrogen). Isolated RNA (1 mg) was first treated with DNase (TaKaRa) in a 10-µl reaction with 5 × gDNA Eraser Buffer (2 µl) and gDNA Eraser (1 µl). The reaction was conducted at 42 °C for 2 minutes.

PrimeScript RT Reagent Kit (TaKaRa) was used for reverse transcription (RT) in a total volume of 20 µl with 4 µl 5 × PrimeScript Buffer, 1 µl PrimeScript RT Enzyme Mix I, 1 µl Oligo dT primer, and 1 µg of the above RNA sample. The mixture was incubated at 37 °C for 15 minutes, and the reaction was inactivated at 85 °C for 5 seconds.

### Real-Time Quantitative PCR

PCR was performed by adding 1 µl cDNA (diluted five times before adding) to the PCR mix containing 5 µl 2 × SYBR Premix Ex Taq, 0.4 µl reverse primers and 0.2 µl ROX Reference Dye. It was carried out with one denature cycle at 95 °C for 10 seconds and 40 amplification cycles at 95 °C for 5 seconds and 60 °C for 30 seconds, followed by a dissociation curve which was constructed at 95 °C for 15 seconds, 60 °C for 15 seconds, and 75 °C for 15 seconds to detect the specificity of the PCR products, on the ABI 7900 real-time PCR system (Applied Biosystems) according to the manufacturer’s protocol. The mRNA levels of target genes were standardized with those of *Gapdh* reference gene and the average fold values were calculated. Primer sequences of PCR for these genes are shown in Table 3. Data (three biological repeats and three technical repeats at each time point) were analyzed by the comparative threshold cycle (CT) method and the standard formula^38^. Each reaction was performed in triplicate.

**Table 3:** Reverse primers for real-time RT-PCR.

Gene (GenBank accession)	Primer Sequences (from 5′ to 3′)	Product size (bp)
*H19*	(Forward) 5′-GCACTAAGTCGATTGCACTGG-3′	163
(NR_001592.1)	(Reverse) 5′-AGGTGCCTGCATCAAGGTGAC-3′	
*Snrpn*	(Forward) 5′-GGATTAGCAGGCCCTGTCAGA-3′	166
(NM_013670.3)	(Reverse) 5′-TGCCTACAGGTGGAGGTGGA-3′	
*Mest*	(Forward) 5′-GTGGTCGGAAGCCCTGAGATAG-3′	112
(NM_008590.2)	(Reverse) 5′-GGGCGATCACTCGATGGAA-3′	
*Peg3*	(Forward) 5′-AAGCCCTTGGGTGTGAGCA-3′	131
(NM_008817.2)	(Reverse) 5′-CCACTTCGGCTCATGTCGTC-3′	
*Gapdh*	(Forward) 5′-TGTGTCCGTCGTGGATCTGA-3′	150
(NM_008084.3)	(Reverse) 5′-TTGCTGTTGAAGTCGCAGGAG-3′	

### Statistical analysis

All computations were performed using SPSS 16.0. Comparisons between the two groups were made using unrelated t-tests and a Pearson chi-square test for BSP. The data are presented as the mean ± SD. In all cases, a values of p < 0.05 were considered statistically significant.

### Data Availability

The datasets generated during or analyzed during the current study are available from the corresponding author on reasonable request.

## Electronic supplementary material

Supplementary Information

## References

[1] de Waal E (2012). Gonadotropin stimulation contributes to an increased incidence of epimutations in ICSI-derived mice. Human Molecular Genetics..

[2] Wen SW (2010). A comprehensive assessment of outcomes in pregnancies conceived by *in vitro* fertilization/intracytoplasmic sperm injection. Eur J Obstet Gynecol Reprod Biol..

[3] Reefhuis J (2009). Assisted reproductive technology and major structural birth defects in the United States dagger. Human Reproduction..

[4] Farhi A (2013). Congenital malformations in infants conceived following assisted reproductive technology in comparison with spontaneously conceived infants. J Matern FetalNeonatal Med..

[5] Tararbit K (2011). Risk of congenital heart defects associated with assisted reproductive technologies: a population-based evaluation. Eur Heart J..

[6] Chang AS, Moley KH, Wangler M, Feinberg AP, DeBaun MR (2005). Association between Beckwith-Wiedemann syndrome and assisted reproductive technology: a case series of 19 patients. Fertil. Steril..

[7] Cox GF (2002). Intracytoplasmic sperm injection may increase the risk of imprinting defects. Am Soc Hum Genet..

[8] Maher ER (2003). Beckwith-Wiedemannsyndrome and assisted reproduction technology (ART). J Med Genet..

[9] Sutcliffe AG (2006). Assisted reproductive therapies and imprinting disorders—a preliminary British survey. Hum Reprod..

[10] Hansen M (2002). The risk of major birth defects after intracytoplasmic sperm injection and *in vitro* fertilization. New England Journal of Medicine..

[11] Katalinic A (2004). Pregnancy course and outcome after intracytoplasmic sperm injection: a controlled, prospective cohort study. Fertility and Sterility..

[12] Zhan QT (2013). An overview of studies on psychological well-being in childrenborn following assisted reproductive technologies. J Zhejiang Univ-Sci B (Biomed & Biotechnol)..

[13] Morgan HD, Santos F, Green K, Dean W, Reik W (2005). Epigenetic reprogramming inmammals. Hum Mol Genet..

[14] Lucifero D, Chaillet JR, Trasler JM (2004). Potential significance of genomic imprintingdefects for reproduction and assisted reproductive technology. Human Reproduction Update..

[15] Rossignol S (2006). The epigenetic imprinting defect of patients with Bechwith-Wiedemann syndrome born after assisted reproductive technology is not restricted to the 11p15 region. J Med Genet..

[16] Zhu JL, Basso O, Obel C, Bille C, Olsen J (2006). Infertility, infertility treatment, and congenital malformations: Danish national birth cohort. BMJ..

[17] Bonduelle M (2005). A multi-centre cohort study of the physical health of 5-year-old children conceived after intracytoplasmic sperm injection, *in vitro* fertilization and natural conception. Hum Reprod..

[18] Kurinczuk J, Bower C (1997). Birth defects in infants conceived by intracytoplasmic sperm injection: an alternative interpretation. Br Med J..

[19] Sakian S (2015). Altered gene expression of H19 and IGF2 in placentas from ART pregnancies. Placenta..

[20] Wang LY (2015). Superovulation Induced Changes of Lipid Metabolism in Ovaries and Embryos and Its Probable Mechanism. PLoSONE..

[21] Wang N (2012). Altered expressions and DNA methylation of imprinted genes in chromosome 7 in brain of mouse offspring conceived from *in vitro* maturation. Reproductive Toxicology..

[22] Wang LY (2013). Alteration of fatty acid metabolism in the liver, adipose tissue, and testis of male mice conceived through assisted reproductive technologies: fatty acid metabolism in ART mice. Lipids in Health and Disease..

[23] de Waal E (2012). Primary epimutations introduced during intracytoplasmic sperm injection (ICSI) are corrected by germline-specific epigenetic reprogramming. PNAS..

[24] Riccio A (2009). Inherited and Sporadic Epimutations at the IGF2-H19 locus in Beckwith-Wiedemann syndrome and Wilms’ tumor. Endocr Dev..

[25] Dekel B (2006). Multiple imprinted and stemness genes provide a link between normal and tumor progenitor cells of the developing human kidney. Cancer Res..

[26] Kanwar YS (2003). Imprinted mesodermal specific transcript (MEST) and H19 genes in renal development and diabetes. Kidney Int..

[27] Schuster AE, Schneider DT, Fritsch MK, Grundy P, Perlman EJ (2003). Genetic and genetic expression analyses of clear cell sarcoma of the kidney. Lab Invest..

[28] Frevel MA, Sowerby SJ, Petersen GB, Reeve AE (1999). Methylation sequencing analysis refines the region of H19 epimutation in Wilms tumor. J Biol Chem..

[29] Palermo GD, Neri QV, Rosenwaks Z (2015). To ICSI or not to ICSI. Semin Reprod Med..

[30] Lazaraviciute G (2014). A systematic review and meta-analysis of DNA methylation levels and imprinting disorders in children conceived by IVF/ICSI compared with children conceived spontaneously. Human Reproduction Update..

[31] Kanber D, Buiting K, Zeschnigk M, Ludwig M, Horsthemke B (2009). Low frequency of imprinting defects in ICSI children born small for gestational age. Eur J Hum Genet..

[32] Chan WE, Hatakeyama C, Robinson WP, Ma S (2011). DNA methylation at *H19*/IGF2 ICR1 in the placenta of pregnancies conceived by *in vitro* fertilization and intracytoplasmic sperm injection. Fertil Steril..

[33] Rancourt RC, Harris HR, Michels KB (2012). Methylation levels at imprinting control regions are not altered with ovulation induction or *in vitro* fertilization in a birth cohort. Hum Reprod..

[34] Fauque P (2010). Modulation of imprinted gene network in placenta results in normal development of *in vitro* manipulated mouse embryos. Hum Mol Genet..

[35] Mann MR (2004). Selective loss of imprinting in the placenta following preimplantation development in culture. Development..

[36] Le F (2013). *In vitro* fertilization alters growth and expression of Igf2/*H19* and their epigenetic mechanisms in the liver and skeletal muscle of newborn and elder mice. Biology of Reproduction..

[37] Li LC, Dahiya R (2002). Meth Primer: designing primers for methylation PCRs. Bioinformatics..

[38] Schefe JH, Lehmann KE, Buschmann IR, Unger T, Funke-Kaiser H (2006). Quantitative real-time RT-PCR data analysis: current concepts and the novel “gene expression’s CT difference” formula. Journal of Molecular Medicine..

