# Supplementary material for: Altered methylations of H19, Snrpn, Mest and Peg3 are reversible by developmental reprogramming in kidney tissue of ICSI-derived mice
Source: Sci Rep. 2017 Sep 20;7:11936. doi: 10.1038/s41598-017-11778-w (PMC5607335; doi:10.1038/s41598-017-11778-w)
Supplement: Supplementary file 1 — Supplementary Information [file 41598_2017_11778_MOESM1_ESM.doc]

**Altered methylations of *H19*, *Snrpn*, *Mest* and *Peg3* are reversible by developmental reprogramming in kidney tissue of ICSI-derived mice**

**Qitao Zhan^1^; Xuchen Qi^2^; Ning Wang^1^; Fang Le^1^; Luna Mao^1^; Xinyun Yang^1^; Mu Yuan^1^; Hangying Lou^1^; Xiangrong Xu^1^; Xijing Chen^1^; Fan Jin^1, 3, *^**

^1^Department of Reproductive Endocrinology, Women’s Hospital, School of Medicine, Zhejiang University, Hangzhou, 310006, China

^2^Department of Neurosurgery, Sir Run Run Shaw Hospital, School of Medicine, Zhejiang University, Hangzhou, 310016, China

^3^Key Laboratory of Reproductive Genetics, Ministry of Education, Hangzhou, 310006, China

*Correspondence address: E-mail: jinfan@zju.edu.cn

**
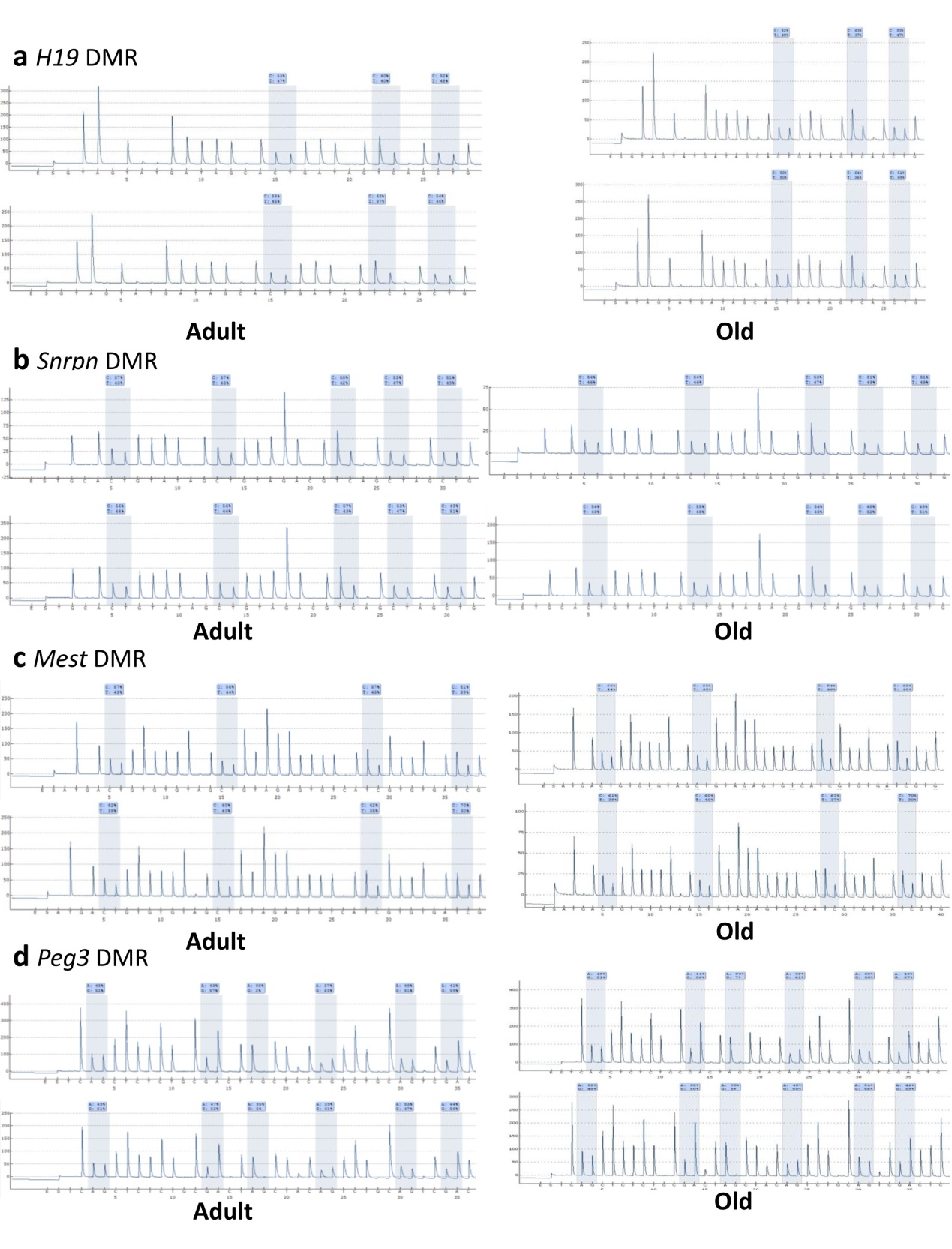
**

**Figure S1** Pyrogram examples of four imprinted gene DMRs. Examples of H19 DMR (a), Snrpn DMR (b), Mest DMR (c) and Peg3 DMR (d) pyrograms produced by pyrosequencing in ICSI-derived mice and TCET conceived mice of adult and old age. The target sequencing for Peg 3 DMR was reversed as a reverse sequencing primer was used for the bisulfite.
